# *POR* polymorphisms are associated with 21 hydroxylase deficiency

**DOI:** 10.1007/s40618-021-01527-2

**Published:** 2021-03-05

**Authors:** F. Pecori Giraldi, S. Einaudi, A. Sesta, F. Verna, M. Messina, C. Manieri, E. Menegatti, L. Ghizzoni

**Affiliations:** 1 Department Clinical Sciences and Community Health, University of Milan, Milan, Italy; 2 Istituto Auxologico Italiano IRCCS, Neuroendocrinology Research Laboratory, Milan, Italy; 3 Department Pediatric Endocrinology, Azienda Ospedaliera Città della Salute e della Scienza, University of Turin, Turin, Italy; 4 Clinical Pathology and Experimental Medicine Unit, Department Clinical and Biological Sciences, University of Turin, Turin, Italy; 5 Division of Endocrinology, Diabetes and Metabolism, Department Medical Sciences, University of Turin, Turin, Italy; 6 Department Medical Genetics, Azienda Ospedaliera Città della Salute e della Scienza, University of Turin, Turin, Italy

**Keywords:** POR, Congenital adrenal hyperplasia, *CYP21A2*, SNP, Genotype–phenotype correlation

## Abstract

**Purpose:**

Genotype–phenotype correlation in congenital 21 hydroxylase deficiency is strong but by no means absolute. Indeed, clinical and hormonal features may vary among patients carrying similar *CYP21A2* mutations, suggesting that modifier genes may contribute to the phenotype. Aim of the present study was to evaluate whether polymorphisms in the p450  oxidoreductase (*POR*) gene may affect clinical features in patients with 21 hydroxylase deficiency

**Methods:**

Sequencing of the *POR* gene was performed in 96 patients with 21 hydroxylase deficiency (49 classic, 47 non-classic) and 43 control subjects.

**Results:**

Prevalence of *POR* polymorphisms in patients with 21 hydroxylase was comparable to controls and known databases. The rs2228104 polymorphism was more frequently associated with non-classic vs classic 21 hydroxylase deficiency (allelic risk 7.09; 95% C.I. 1.4–29.5, *p* < 0.05). Classic 21 hydroxylase-deficient carriers of the minor allele in the rs2286822/rs2286823 haplotype presented more frequently the salt-wasting form (allelic risk 1.375; 95% C.I. 1.138–1.137), more severe Prader stage at birth (allelic risk 3.85; 95% C.I. 3.78–3.92), higher ACTH levels, and younger age at diagnosis.

**Conclusions:**

Polymorphisms in the *POR* gene are associated with clinical features of 21 hydroxylase deficiency both as regards predisposition to classic vs non-classic forms and severity of classic adrenal hyperplasia.

**Supplementary Information:**

The online version contains supplementary material available at 10.1007/s40618-021-01527-2.

## Introduction

Congenital adrenal hyperplasia due to 21 hydroxylase deficiency ranks among the most frequent endocrine genetic defects, and considerable efforts have been expended to understand its pathophysiology and ameliorate diagnostic and therapeutic strategies. Clinical presentation depends on the severity of the underlying enzyme defect and, in patients who are compound heterozygotes for *CYP21A2* mutations, phenotype usually correlates with the less severely mutated allele and residual 21 hydroxylase activity [1]. However, genotype–phenotype correlation is by no means absolute [1–3]. In fact*,* a recent study on over 1500 families demonstrated that direct genotype–phenotype correlation could be observed in less than 50% of *CYP21A2* genotypes [4]. Furthermore, sib pairs carrying identical *CYP21A2* mutations but presenting different phenotypes, i.e., salt-wasting, simple virilizing or even only mild hyperandrogenism, have also been reported [5, 6].

It is conceivable that other factors may influence 21 hydroxylase enzyme activity and contribute to the variability of clinical features among different enzyme defects. P450 oxidoreductase (POR), a flavoprotein involved in several microsomal reactions including 21 hydroxylation [7], is one of the most likely candidates. Interest in POR stems for the fact that mutations in *POR* lead to deranged steroidogenesis with sexual ambiguity and cortisol deficiency [8, 9]. In addition to these enzyme-disrupting mutations, the *POR* gene is highly polymorphic and variants have been associated with altered 21 hydroxylase and 17 hydroxylase/17,20 lyase activity [7].

Aim of the present study is to evaluate whether genomic variants in *POR* contribute to the phenotype of patients with congenital adrenal hyperplasia due to *CYP21A2* mutations. Associations between polymorphisms and clinical features will aid in the understanding of 21 hydroxylase defects pathophysiology and, hopefully, lead to the development of individualized treatment strategies.

## Methods

### Patients

Ninety-six Caucasian patients with classic (49 patients, 28 females, 21 males) and non-classic (47 patients, 21 females, 26 males) congenital adrenal hyperplasia due to 21 hydroxylase deficiency were studied over the past 2 decades at the Azienda Ospedaliera Città della Salute e della Scienza in Turin. Data relative to presentation and treatment were collected for statistical analysis. Hormonal and clinical assessments were performed as described in [3, 10]. Age at diagnosis was 15.6 ± 1.71 days in patients with classic adrenal hyperplasia and 7.93 ± 0.63 for pediatric and 37.4 ± 3.1 years for adult patients with non-classic 21 hydroxylase deficiency, respectively*.* Patients with classic adrenal hyperplasia were classified as simple virilising or salt-wasting based on clinical and biochemical signs, e.g., dehydration, failure to thrive, hyponatriemia, and hyperkalemia. Salt wasting was present in 85% of patients with classic 21 hydroxylase deficiency (77% of females and 95% of males, N.S.), whereas hypoglycemia was detected in 8%. Among patients with the non-classic form, premature pubarche was present in 20 (19 females and 1 male), precocious puberty in 7 (all females), hirsutism alone in 9 females (43%), and acne and hirsutism in 5 females (24%). The remaining patients were asymptomatic relatives of patients, detected while performing family studies. In parallel, 43 subjects, 22 women and 21 men, all wild-type for *CYP21A2*, were used as controls for *POR* sequencing.

### *CYP21A2* sequencing

Total genomic DNA was extracted from leukocytes collected in EDTA-coated tubes, using the QIAamp DNA Blood Midi Kit (Qiagen, Milan, Italy), according to the manufacturer’s instructions. Gene deletions and large gene conversion were evaluated by Southern blotting analysis with *TaqI* digestion, whereas the most common and pseudogene-derived point mutations were detected by multiplex minisequencing using *CYP21A2*-specific long-range PCR as template (reference sequence NM_000500.9). Chimeric genes were identified by long-range PCR using appropriate, specific primers for *CYP21P/CYP21* and *CYP21/CYP21P* chimeras, respectively. Obtained amplicons were further characterized by multiplex minisequencing mutation analysis [10].

Patients with classic 21 hydroxylase deficiency carried 11 different *CYP21A2* mutations with 20% presenting the deletion/c.*13G>A genotype, 16% the deletion/I172N genotype, 12% the homozygous c.*13G>A genotype, 8% the c.*13G>A/Q318X genotype, and 6% the deletion/R356W genotype (further details are shown in supplementary Table 1; https://doi.org/10.13130/RD_UNIMI/DNZMA0). Among patients with non-classic adrenal hyperplasia, 36% presented the V281l/V281L genotype, 17% the V281L/P453S genotype, and 23% the V281L/I172N and V281L/deletion genotypes (further details are shown in Supplementary Table 1). *CYP21A2* mutations were grouped according to severity of the enzyme defect [5, 11]: no residual activity = group O (deletion, gene conversion, Q318X, R356W), approx. 1% residual activity = group A (c.*13G>A,N381I), 2–3% residual activity = group B (I172N, R341P), and > 30% residual activity = group C (V281L, P30L, P453S, L288F). Patients were categorized according to the less severe mutation group [11]. As expected, patients with classic adrenal hyperplasia were mostly group O (18%), A (44%), and B (30%), whereas patients with non-classic adrenal hyperplasia were group C (supplementary Table 1).

**Table 1:** *POR* polymorphisms detected in patients with 21 hydroxylase deficiency and control subjects

SNP	Location	MAF	In silico	Prevalence in controls			HWE	Prevalence in classic CAH			Prevalence in non-classic CAH			Classic vs non-classic
				wt	het	homo	*p*	wt	het	homo	wt	het	homo	
rs1135612	exon 5 A/G	0.24	synon	51	35	14	0.21	59	31	10	70	26	4	N.S
rs41301394§	intron 8 C/T	0.29		56	37	7	0.88	47	39	14	53	43	4	N.S
rs4732516	intron 10 G/C	0.06		88	12	0	0.68	94	4	2	85	15	0	N.S
rs2286822*	intron 11 C/T	0.30		42	44	14	0.78	45	47	8	62	30	8	N.S
rs2286823*	intron 11 G/A	0.31		44	45	14	0.78	34	52	14	62	31	7	N.S
rs2228104	exon 13 C/T	0.06	synon	88	12	0	0.68	96	4	0	77	23	0	*p* < 0.05
rs1057868§	exon 13 C/T	0.28	A503V	53	37	10	0.62	51	35	14	59	38	3	N.S
rs1057870	exon 14 G/A	0.32	synon	49	44	7	0.64	49	45	6	40	38	22	N.S

Prevalence is reported in %

SNPs in LD are identified by * (block 1) and § (block 2)

*MAF* minor allele frequency, *synon* synonymous variant, *wt* wild-type, *het* heterozygous carriers, *homo* homozygous carriers, *HWE* Hardy–Weinberg Equilibrium

### *POR* sequencing

Primers specific for *POR* (NG_023211.1, 71,754 bp; exons 1–15) were designed using Primer3 [12] to include at least 100 bases of flanking intronic DNA. PCR amplification with 100 ng genomic DNA and GoTAq DNA Polymerase (Promega, Madinson WI) was performed at high touchdown cycling conditions (14 cycles with 0.5° C decrease at each cycle from 65 °C to 58.5 °C, starting with 95 °C for 5 min) followed by 25 cycles at 58 °C annealing. *POR* sequences were compared to reference ENSG00000127948.9 and variants identified. Variants were searched on EntrezGene, Human Gene Mutation Database (HGMD), Human Genome Variation Database (HGVbase), Ensembl, and dbSNP. Significance of identified variants was assessed using Mutation taster [13], Mutation assessor [14], SIFT [15], and FATHMM [16]. Sequence variants were interpreted according to the American College of Medical Genetics and Genomics and the Association for Molecular Pathology 2015 recommendation [17].

### Statistics

Variant distribution and deviation from Hardy–Weinberg equilibrium were assessed in controls. Linkage disequilibrium (i.e., D’ and *r*^2^) and haplotype frequencies were computed by Haploview 4.1, Broad Institute, Cambridge, MA [18]. Association between allelic risk and dichotomic phenotypic features was first tested by Pearson’s chi squared test assuming no model effect and allelic/genotypic risk calculated; significant associations were subsequently tested by Score test for dominant effect model, in view of summary data. Cochrane–Armitage test for trend was used for ordered categorical variables. Mann–Whitney or Kruskal–Wallis ANOVA were used to determine differences in continuous parameters between variant/haplotype carriers and wild-type. Furthermore, logistic regression analysis was used to assess associations between single-nucleotide polymorphisms (SNP) and continuous variables taking covariates such as sex into account. Bonferroni’s correction for multiple comparisons was applied where appropriate. The sample size allowed detection of OR > 3.0 with at least 80% potency, given minor allele frequency (MAF) greater than 5% and 0.05 two-sided alfa [19]. Data are reported as mean ± S.E.M. or Odds ratio (O.R.) with 95% confidence interval (95% C.I.)

## Results

### *POR* polymorphisms

Eight different known *POR* single-nucleotide polymorphisms with MAF greater than 5% were identified (see Table 1). The four intronic variants are benign as assessed by the above-mentioned pathogenicity prediction software; as regards proximity to intron–exon boundaries, rs41301394 is located 35 bp upstream to exon 9, rs4732516 is located 13 bp upstream to exon 11, and rs2286822 and rs2286823 are located 12 and 20 bp, respectively, downstream to exon 11. Variants rs1135612, rs2228104, and rs1057870 are synonymous SNPs (rs1135612: p.P129 = , rs2228104: p.A485 = ; rs1057870: p.s572 =), whereas rs1057868 is a missense p.A503V variant (previously reported as POR*28). Additional intronic (rs371932012, rs72557932, and rs72557956) and exonic SNPs (rs10262966, rs41295381, rs1585129713, rs11540674, rs72557941, rs377500167, rs150414675, rs370823127, rs375387233, rs1320059073, and rs145782750) occurred in individual patients or controls. MAF for all SNPs corresponded to data reported in available databases (www.ncbi.nlm.nih.gov/snp). The eight major SNPs were in Hardy–Weinberg equilibrium in controls and no differences in sex distribution were observed in either patients or controls.

Two haplotype blocks were identified, each comprising two SNPs (Supplemental Fig. 1; https://doi.org/10.13130/RD_UNIMI/DNZMA0): rs2268622 and rs2268623 with *r*^2^ 0.966 and D’ 1.0 (95% C.I. 0.96–1.0), while rs2057868 and rs41301394 presented *r*^2^ 0.88 and D’ 0.963 (95% C.I. 0.90–0.99).

### Impact of* POR* polymorphisms on prevalence of classic and non-classic 21 hydroxylase deficiency

Prevalence of the rs2228104 polymorphism was significantly higher among patients with non-classic compared with patients with classic 21 hydroxylase deficiency (11.7% vs 2.0%, *p* < 0.05, see Table 1 for genotype frequency) and the allelic risk associated with non-classic CAH was equal to 7.09 (95% C.I. 1.37–29.52, *p* < 0.05). In keeping, the T allele was present in 11.0 vs 2.17% (*p* < 0.05) of patients with group C mutations and allelic risk associated with group C was 5.91 (95% C.I. 1.19–25.81, *p* < 0.05; Supplemental Table 2).

None of the other *POR* polymorphisms or haplotypes were associated with increased risk of either classic or non-classic 21 hydroxylase deficiency (Table 1) or *CYP21A2* defect grouping (Supplemental Table 2).

### Impact of *POR* polymorphisms on clinical features of classic and non-classic 21 hydroxylase deficiency

**Fig. 1:**
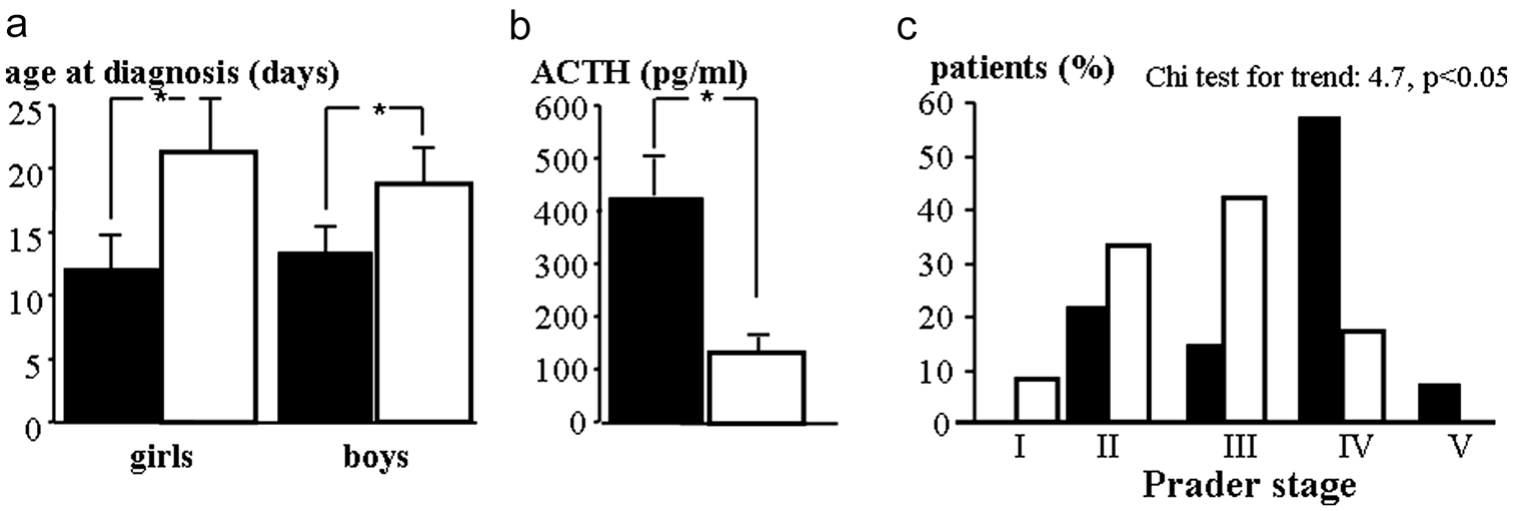
Age at first dosing, ACTH levels, and Prader stage according to *POR* rs2268622/23 haplotype. **a** Age at diagnosis in girls and boys carrying minor (black) or wild-type (white) *POR* rs2268622/23 alleles. **b** Plasma ACTH levels in children carrying minor (black) or wild-type (white) alleles. **c** Severity of Prader stage at birth in girls carrying minor (black) or wild-type (white) alleles.*p < 0.05

Significant differences as regards clinical presentation in patients with classic congenital hyperplasia were observed according to the *POR* rs2268622/23 haplotype. In fact, carriers of the minor alleles were significantly younger at diagnosis (11.8 ± 1.37 vs 20.0 ± 4.03 days, *p* < 0.05) (Fig. 1) and presented higher plasma ACTH levels (416.8 ± 82.81 vs 148.6 ± 18.43 pg/ml, *p* < 0.05, Fig. 1) compared to children with classic CAH carrying the wild-type CC/GG alleles. Logistic regression confirmed association between the rs2268622/23 haplotype and age (coefficient -0.08, p < 0.05) and ACTH levels at diagnosis (coefficient 0.10, *p* < 0.05), regardless of sex of the patient. Indeed, age at diagnosis was comparable among males and females wild-type for the *POR* wild-type CC/GG alleles (19.1 ± 2.72 vs 21.4 ± 2.31 days, respectively, N.S.) and carriers of the rs2268622/23 haplotype (13.5 ± 1.65 vs 12.4 ± 2.17 days, respectively, N.S.). Furthermore, 96% of minor allele carriers presented salt-wasting at birth (Score test for dominant model 6.29, *p* < 0.01; allelic risk 1.375; 95% C.I. 1.138–1.137). In addition, Prader stage was more severe among girls carrying the minor vs wild-type alleles (Chi-square for trend 4.7, *p* < 0.05, Fig. 1) as was the risk for severe (i.e., stages IV and V) vs mild Prader stage (i.e., stages I, II, and III) at birth (score test for dominant model 6.0, *p* < 0.05; allelic risk 3.85; 95% C.I. 3.78–3.92). The rs2268622/23 haplotype was equally distributed among different *CYP21A2* defect groups: 70%, 62%, and 53%, respectively, among group O, A, and B (Chi-square 4.96 with 2 d.f., N.S.); none of the 3 patients in group C carried the rs2268622/23 haplotype.

In patients with non-classic 21 hydroxylase deficiency, no differences in phenotypic features could be detected between carriers of the *POR* rs2268622/23 haplotype and wild-type patients*.* None of the other *POR* polymorphisms or the 868/194 haplotype were associated with clinical features of classic or non-classic 21 hydroxylase deficiency.

## Discussion

The present study shows that polymorphisms in the *POR* gene are associated with the phenotype of patients with congenital adrenal hyperplasia, a novel finding in the pathophysiology of 21 hydroxylase deficiency.

POR (cytochrome P450 oxidoreductase) is a flavoprotein which subserves several microsomal enzymatic reactions, most notably those deputized to drug metabolism and steroid biosynthesis [20]. In the context of adrenal steroidogenesis, POR contributes to 21 hydroxylase, 17 hydroxylase, and 17,20 lyase activity; indeed, mutations in *POR* may lead to sexual ambiguity and adrenal insufficiency, i.e., Antley–Bixler syndrome with genital abnormalities and disordered steroidogenesis (OMIM 201750) [9, 21, 22]. Urinary steroid profile usually indicates combined CYP21 and CYP17 deficiency [9, 21, 23], although the extent to which either enzyme is compromised varies according to the site of the *POR* mutation: the A287P variant is associated with preferential inhibition of 17 alpha hydroxylase and 17,20 lyase activity [21, 24], whereas variants located in the electron transfer area, e.g., Y181D, H628P, lead to equal impairment of CYP17 and CYP21 activities [24].

In contrast to these rare, enzyme-disrupting mutations, polymorphisms in the *POR* gene occur in up to one-third of the population and are associated with altered *POR* transcription and enzymatic activity [25–27]. In fact, both increased and reduced POR activity has been observed in liver samples from individuals carrying different *POR* variants [28] and, furthermore, the impact of *POR* polymorphisms appears CYP substrate-specific [29].

Given the high frequency of *POR* variants and its impact on CYP enzyme function, we hypothesized that *POR* may act as modifier gene, thereby contributing to the variability in genotype–phenotype correlation among patients with defects in 21 hydroxylase activity. Interestingly, a patient with coexisting *POR* and *CYP21A2* defects had been described some 30 years ago [30]; genotyping revealed c.293-13A > G/P30L mutations on *CYP21A2* and a A287P *POR* variant on the maternal allele—the most frequent variant among Caucasians [31]. Clinicians were struck by the mild, non-progressive virilization in context of severe, salt-wasting 21 hydroxylase deficiency suggesting an impact of the heterozygous *POR* defect on clinical features of congenital adrenal hyperplasia.

We genotyped *POR* and identified eight variants spread across the *POR* gene, both intronic and exonic. All variants had been previously described and variant assessment by in silico predictive programs as well as literature searches allowed classification of intronic SNPs, i.e., rs41301394, rs4732516, rs2286822, and rs2286823, as well as the three synonymous SNPs, i.e., rs1135612, rs2228104, and rs1057870, as benign [17]. Conversely, the rs1057868 polymorphism leads to an alanine-to-valine switch (i.e., p.A503V) and has been the focus of several studies under the moniker POR*28. Studies in Asian as well as other populations have revealed an association of the variant with bladder cancer [32], post-kidney transplant diabetes [33], and hepatitis B virus-related hepatocellular carcinoma [20]. A small study evaluated p.A503V in 17 patients with either classic or non-classic adrenal hyperplasia [34], but was clearly underpowered to detect any association. This same study did not detect differences in normal 21 hydroxylase activity in vitro between wild-type and p.A503V *POR* transfectants; the effect of this polymorphism in mutated *CYP21A2* remains to be established. Of note, *POR* variants have been mostly studied for their effect on CYP17 rather than CYP21 activity [22, 24].

Our data now provide evidence for the association between *POR* polymorphisms and clinical features of congenital adrenal hyperplasia, thereby establishing *POR* as a genetic modifier of *CYP21A2* defects. *POR* polymorphisms appear to act both as primary and secondary genetic modifiers as the rs2228104 minor allele is associated with higher prevalence of non-classic adrenal hyperplasia and the rs2268622/rs2268623 minor alleles are associated with severity of clinical features in classic adrenal hyperplasia.

The rs2228104 polymorphism is a synonymous alanine variant on exon 13 (i.e., p.A485 =), whose impact is as yet unknown. It lies within the FAD moiety and an alanine-to-threonine variant at the same amino acid (p.A485T) is associated with reduced 17 hydroxylase/17,20 lyase activity [35]. A single study has so far evaluated the rs2228104 polymorphism and reported no changes in POR synthesis or activity in liver specimens from individuals carrying different alleles [28]. Interestingly, minor allele frequency is low, i.e., 0.03–0.1%, especially in Europeans, as shown in databases, e.g., Genome Aggregation Database, Broad Institute, and individuals studies including our own [27, 28] and, in fact, homozygous carriers of the minor allele (TT) appear to be extremely rare, even absent in some series [27, 28]. In our study, frequency of the minor allele among patients with non-classic adrenal hyperplasia was higher than expected, i.e., 11%, and was associated with a sixfold higher risk of being affected by non-classic rather than classic congenital adrenal hyperplasia. The effect of rs2228104 is reminiscent of primary modifier genes in thalassemia; indeed, *KLF1* (Kruppel-like factor 1) variants are overrepresented among patients with thalassemia intermedia compared to thalassemia major [36]. Given that the *POR* gene is located on chromosome 7 and *CYP21A2* on chromosome 6, linkage disequilibrium between rs2228104 and *CYP21A2* mutations associated with non-classic 21 hydroxylase deficiency appears unlikely. It is tempting to speculate that this finding could be due to genetic drift or selection, if rs2228104 were to confer a selective advantage to the non-classic phenotype.

As regards the rs2268622/rs2268623 haplotype, our results showed an association with severity of clinical features among children with classic congenital adrenal hyperplasia. Newborns with the minor alleles presented a more severe presentation with greater prevalence of salt-wasting and higher Prader stage in girls. This finding is of particular interest given that it reflects disease severity at presentation. In line with this evidence, higher plasma ACTH concentrations, albeit a measurement subject to considerable variability [37], and younger age at diagnosis were observed in carriers of the minor alleles. In our study, the dominant model proved statistically significant, indicating that the minor T/A alleles are associated with a more severe clinical presentation. In vitro studies on the rs2268622/rs2268623 alleles reported reduced POR protein and activity in liver samples from minor TT and AA genotypes [28], thus, although intronic, these sequence variants impact POR function. Our findings suggest that *POR* rs2268622/rs2268623 are associated with greater impairment of CYP21 activity, thus more severe clinical presentation. In support of this hypothesis, the polymorphism was equally prevalent across severity of *CYP21A2* mutation groups associated with classic congenital hyperplasia. Studies on the effect of this haplotype on 21 hydroxylase activity, with focus on severe enzymatic impairments such as those occurring with mutations leading to classic 21 hydroxylase deficiency [38], are needed to substantiate this hypothesis.

Congenital adrenal hyperplasia due to 21 hydroxylase deficiency is a severe endocrine genetic defect with good though not absolute genotype–phenotype correlation [1–3]. In fact*, *in vitro behavior of *CYP21A2* mutations was not wholly predictive of the clinical phenotype [5] and attempts to predict the phenotype based on *CYP21A2* mutations fall short of reliable prediction [11]. Premature pubarche and hirsutism, for example, are associated with different percentages of both severe and mild *CYP21A2* mutations [39, 40]. Furthermore, a recent large-scale study reported that less than 50% of *CYP21A2* genotypes presented the predicted phenotype [4] and confirmed evidence [5, 6], indicating that while some mutations are predominantly associated with either the salt-wasting or the simple virilizing phenotype, they may give rise to other disease phenotypes as well. *POR* polymorphisms may contribute this variability in clinical presentation with a given *CYP21A2* defect, thus act as a secondary modifier gene [41], much like it occurs in other genetic disorders. One well-known example of such modifier genes is *TGFB1* in cystic fibrosis with specific variants associated with greater disease severity [42].

In conclusion, our study demonstrated that *POR* acts as a modifier gene in congenital adrenal hyperplasia due to 21 hydroxylase deficiency. *POR *polymorphisms are associated with predisposition to classic vs non-classic forms as well as severity of classic adrenal hyperplasia. *POR* polymorphisms may represent the basis of a more individualized approach to management of this disease.

## Supplementary Information

Below is the link to the electronic supplementary material.

Supplementary file1 (DOC 47 KB)

Supplementary file2 (DOC 44 KB)

Supplementary file3 (TIF 94 KB)

## Data Availability

Datasets generated during the current study are available at ClinVar repository https://submit.ncbi.nlm.nih.gov/subs/clinvar_org/SUB7932923/
