# Supplementary material for: POR polymorphisms are associated with 21 hydroxylase deficiency
Source: J Endocrinol Invest. 2021 Mar 5;44(10):2219–26. doi: 10.1007/s40618-021-01527-2 (PMC8421294; doi:10.1007/s40618-021-01527-2)
Supplement: Supplementary file 1 — Supplementary file1 (DOC 47 KB) [file 40618_2021_1527_MOESM1_ESM.doc]

**Supplemental Table 1. *CYP21A2* genotypes identified in patients with 21 hydroxylase deficiency**

Non Classic Adrenal hyperplasia

| **genotypes** | **%** | **group** |
| --- | --- | --- |
| V281L/V281L | 36.2 | C |
| V281L/P453S | 17.0 | C |
| V281L/large deletion | 14.9 | C |
| V281L/I172N | 8.5 | C |
| V281L/c.*13G>A | 8.5 | C |
| V281L/Q318X | 6.4 | C |
| P30L/c.*13G>A | 4.2 | C |
| V281l/L288F | 2.1 | C |
| P30L/large deletion | 2.1 | C |

Classic Adrenal hyperplasia

| **genotypes** | **%** | **group** |
| --- | --- | --- |
| large deletion/c.*13G>A | 20.4 | A |
| large deletion/I172N | 16.3 | B |
| homozygous c.*13G>A | 12.2 | A |
| c.*13G>A/Q318X | 8.2 | A |
| large deletion/R356W | 6.1 | O |
| c.*13G>A/I172N | 6.1 | B |
| P30L/N387I | 4.1 | C |
| Q318X/del 8bp | 4.1 | O |
| Q318X/I172N | 4.1 | B |
| homozygous large deletion | 2.0 | O |
| c.*13G>A/del 8bp | 2.0 | A |
| c.*13G>A/R356W | 2.0 | A |
| R356W/del 8bp | 2.0 | O |
| large deletion/del8bp | 2.0 | O |
| large deletion/Q318X | 2.0 | O |
| R342P/P920insT | 2.0 | B |
| Q318X/P453S | 2.0 | C |
| R342P/R356W | 2.0 | B |
