# Supplementary material for: POR polymorphisms are associated with 21 hydroxylase deficiency
Source: J Endocrinol Invest. 2021 Mar 5;44(10):2219–26. doi: 10.1007/s40618-021-01527-2 (PMC8421294; doi:10.1007/s40618-021-01527-2)
Supplement: Supplementary file 2 — Supplementary file2 (DOC 44 KB) [file 40618_2021_1527_MOESM2_ESM.doc]

**Supplemental Table 2. *POR* polymorphisms according to *CYP21A2* genotype group**

| **SNP** | **prevalence in**  **group 0** | | | **prevalence in**  **group A** | | | **prevalence in**  **group B** | | | **prevalence in**  **group C** | | |
| --- | --- | --- | --- | --- | --- | --- | --- | --- | --- | --- | --- | --- |
|  | **wt** | **ht** | **ho** | **wt** | **ht** | **ho** | **wt** | **ht** | **ho** | **wt** | **ht** | **ho** |
| rs1135612 | 56 | 33 | 11 | 54 | 32 | 14 | 62 | 31 | 7 | 72 | 24 | 4 |
| rs41301394§ | 44 | 45 | 11 | 59 | 32 | 9 | 31 | 44 | 25 | 52 | 42 | 6 |
| rs4732516 | 100 | 0 | 0 | 86 | 9 | 5 | 100 | 0 | 0 | 86 | 14 | 0 |
| rs2286822# | 33 | 56 | 11 | 41 | 50 | 9 | 50 | 44 | 6 | 64 | 28 | 8 |
| rs2286823# | 33 | 56 | 11 | 36 | 55 | 9 | 50 | 44 | 6 | 64 | 28 | 8 |
| rs2228104 | 100 | 0 | 0 | 91 | 9 | 0 | 100 | 0 | 0 | 78 | 22 | 0 |
| rs1057868§ | 44 | 44 | 12 | 64 | 27 | 9 | 37 | 38 | 25 | 58 | 38 | 4 |
| rs1057870 | 44 | 56 | 0 | 50 | 41 | 9 | 56 | 38 | 6 | 40 | 40 | 20 |

Prevalence is reported in %.; wt: wild-type; ht: heterozygous carriers, ho: homozygous carriers.

SNPs in LD are identifed by # (block 1) and § (block 2).
