# Supplementary figures and images for: POR polymorphisms are associated with 21 hydroxylase deficiency
Source: J Endocrinol Invest. 2021 Mar 5;44(10):2219–26. doi: 10.1007/s40618-021-01527-2 (PMC8421294; doi:10.1007/s40618-021-01527-2)

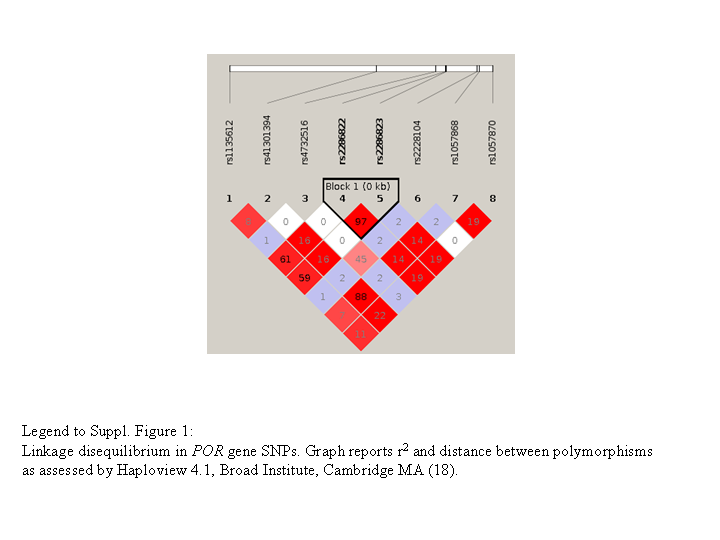

Supplement: Supplementary file 3 — Supplementary file3 (TIF 94 KB) [file 40618_2021_1527_MOESM3_ESM.tif]
